# Supplementary material for: Interplay between α2-chimaerin and Rac1 activity determines dynamic maintenance of long-term memory
Source: Nat Commun. 2019 Nov 22;10:5313. doi: 10.1038/s41467-019-13236-9 (PMC6876637; doi:10.1038/s41467-019-13236-9)
Supplement: Supplementary file 1 — Supplementary Information [file 41467_2019_13236_MOESM1_ESM.pdf]

Supplemental Information

Interplay between α2-chimaerin and Rac1 activity determines dynamic maintenance of long-term memory

Li Lv et al. Nature Communications. 2019

Supplemental Figures

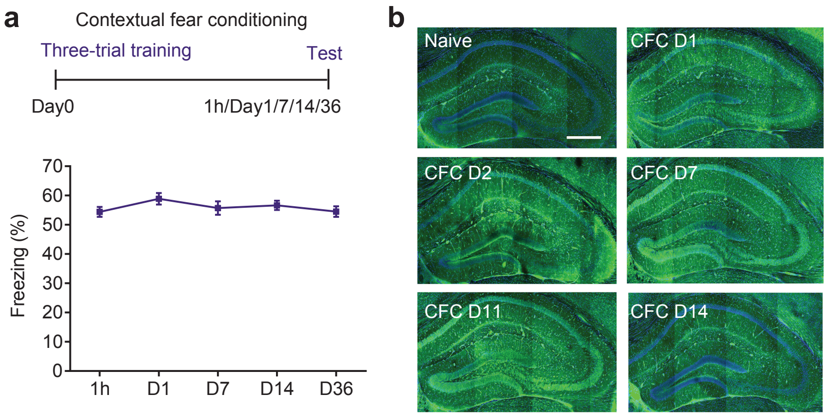

Supplementary Figure 1. A stable contextual fear memory after three-trial CFC and the hippocampal Rac1 activity after single-trial CFC. **a** Behavioral schedule for three-shock CFC-induced memory retention curve (D, day) in wild-type mice. n = 8, 48, 35, 45, 29 mice (one-way ANOVA). **b** Images showing the Rac1 activity in the hippocampal CA1 region of both Na mice and CFC mice at days 1, 2, 7, 11 and 14 after CFC. Brain sections were stained for Rac1-GTP (green) and for all neuronal nuclei (blue). Data are presented as means ± SEM. Also see Supplementary Table 1.

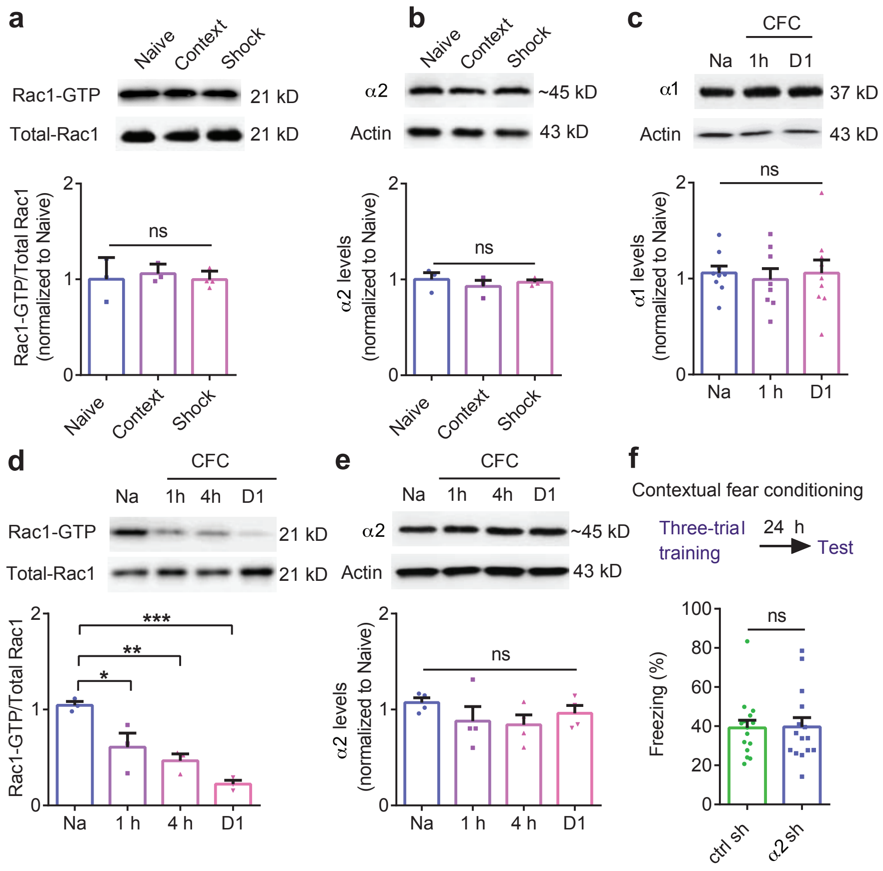

Supplementary Figure 2. Representative image of western blots (a-e) and the effects of α2-chimaerin knockdown on three-trial CFC (f). **a** and **b** Western blots assays showing the levels of Rac1 activity (anti-Rac1-GTP), total Rac1, α2-chimaerin and actin in the hippocampal extracts from both naive mice and trained mice, with only context or only foot shock. $n = 3, 3, 4$ mice (a), $n = 3, 3, 3$ mice (b) (one-way ANOVA). **c** Levels of α1-chimaerin and actin in the hippocampal extracts from both naive (Na) and trained (one shock) mice at various retention intervals (1 h and day 1). $n = 9, 8, 9$ mice (one-way ANOVA). **d** Levels of Rac1 activity (anti-Rac1-GTP) and total Rac1in the hippocampal extracts from both naive (Na) and trained (three shocks) mice at various retention intervals (1 h, 4 h and day 1). $^*P < 0.05$, $^{**}P < 0.01$, and $^{***}P < 0.001$ (from Unpaired t-test); $n = 3, 3, 3, 3$ mice. **e** Levels of α2-chimaerin and actin in the hippocampal extracts from both naive (Na) and trained (three shocks) mice at various retention intervals (1 h, 4 h and day 1). $n = 3, 3, 3, 3$ mice (one-way ANOVA). **f** α2-chimaerin

knockdown had no effects on three-shock CFC-induced fear memory. n = 15, 15 mice (Unpaired t-test). Data are presented as means ± SEM. Also see Supplementary Table 1.

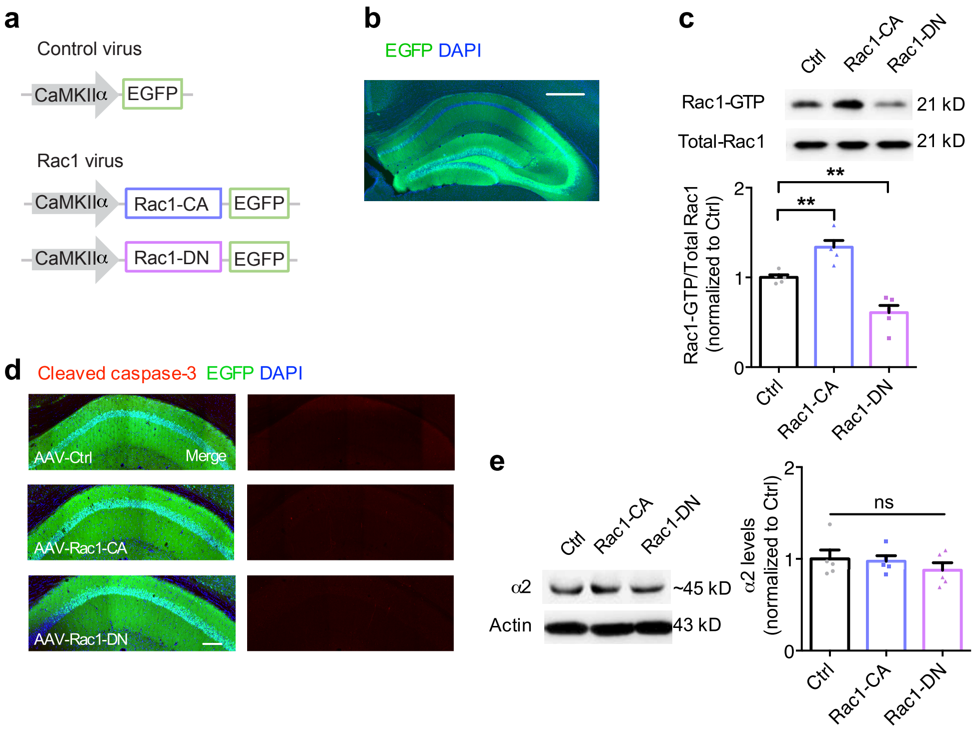

Supplementary Figure 3. The levels of Rac1 activity and cleaved caspase-3 in the hippocampus. **a** Construct of AAVs. Diagram of AAV expressing EGFP alone (Ctrl), and either the constitutively active form of Rac1 (Rac1-CA) or dominant-negative form of Rac1 (Rac1-DN) fused with EGFP under the control of the calcium/calmodulin-dependent protein kinase IIα (CaMKIIα) promoter. **b** Coronal sections of the dorsal hippocampus following viral injections (bottom). Brain sections were stained for all cell nuclei (blue). Scale bar, 500 μm. **c** Representative western blots (top) and data (bottom) presenting the levels of both total Rac1 and Rac1 activity in the hippocampus following viral injections. $^{**}P < 0.01$ (from one-way ANOVA); n = 5, 5, 5 mice. **d** Coronal sections of the hippocampus with immunostaining of cleaved caspase-3 (red) following the viral manipulations of Rac1 activity with Rac1-DN or Rac1-CA. Scale bar, 100 μm. **e** Representative western blots (top) and data (bottom) presenting the levels of α2-chimaerin and actin in the hippocampus following viral injections. n = 5, 5, 5 mice (one-way ANOVA). Data are presented as means ± SEM. Also see Supplementary Table 1.

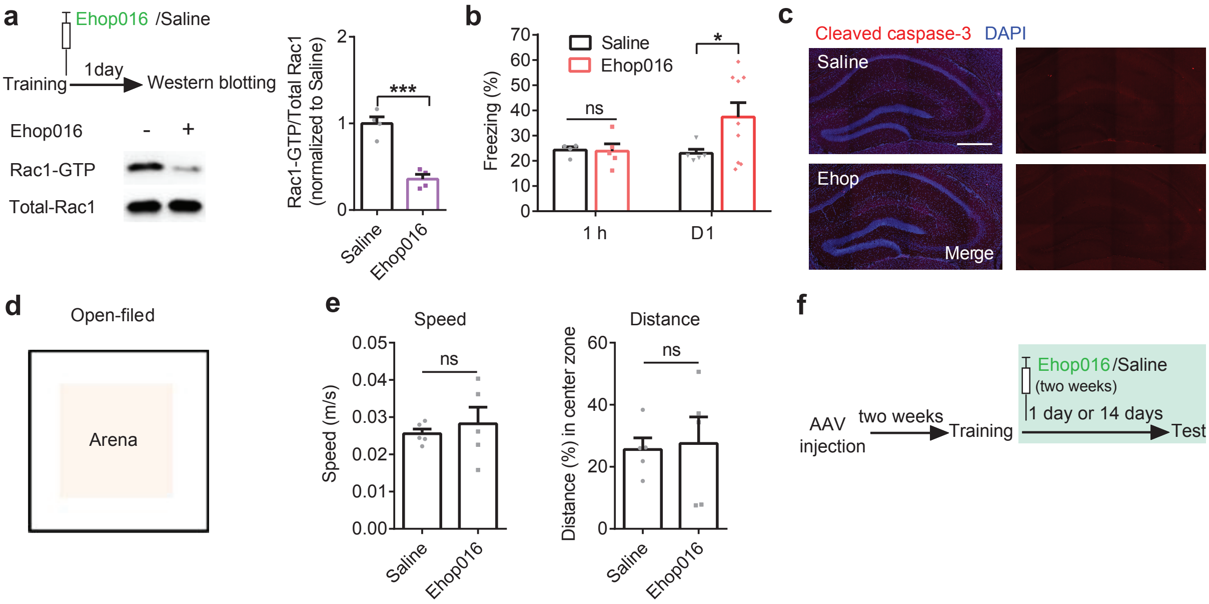

Supplementary Figure 4. The effects of Ehop016 injection on the levels of hippocampal Rac1 activity, cleaved caspase-3 and the basic parameters of mice behavior. **a** Depiction of the experimental design (top) and data (bottom) showing the levels of both Rac1 activity and total Rac1 in mice after Ehop016 injection. Mice were administered with either Ehop016 (intraperitoneally [i.p.] 20 mg/kg) or saline (i.p.) immediately after one-shock CFC. $^{***}P < 0.001$ (from Unpaired t-test); $n = 4, 4$ mice. **b** Behavioral effects of the Rac1 inhibitor Ehop016 on 1 h and 24 h contextual fear memory. $^{*}P < 0.05$ (from Unpaired t-test); $n = 4, 5, 5, 9$ mice. **c** Coronal sections of the hippocampus with immunostaining of cleaved caspase-3 (red) following Ehop016 injections for one week. Scale bar, 500 µm. **d** and **e** The mice were placed in an open field (d) to test the basal locomotor activity, as indicated by both the speed and distance in the arena (e), and no significant difference was observed between the groups. Light red shading signifies the arena. $n = 5, 5$ mice (Unpaired t-test). **f** Representation of the experimental design. Two weeks after AAV injection, both AAV-ctrl shRNA mice and AAV-α2 shRNA mice were trained using one-shock CFC. Subsequently, they were injected with Ehop016 (intraperitoneally [i.p.] 20 mg/kg) or saline (i.p.), once a day, for two weeks. Finally, the test was performed at days 1 and 14. Data are presented as means ± SEM. Also see Supplementary Table 1.

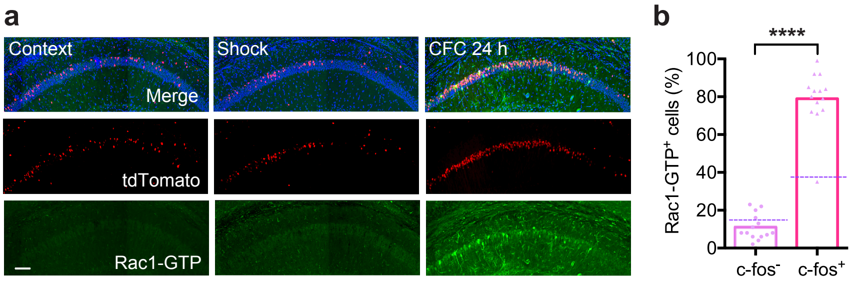

Supplementary Figure 5. The distribution and percentages of Rac1 activation in CA1. **a** Coronal section of CA1 engram cells (tdTomato) labeling with anti-Rac1-GTP (green) in response to context only, shock only and CFC. Scale bar, 100 μm. **b** Percentages of CA1 engram cells and non-engram cells labeling with anti-Rac1-GTP. Chance levels, indicated by grape dashed lines, were estimated at 15.7% (c-fos$^-$) and 38.2% (c-fos$^+$). ****$P < 0.0001$ (from Unpaired t-test); n = 14, 14 mice. Data are presented as means ± SEM. Also see Supplementary Table 1.

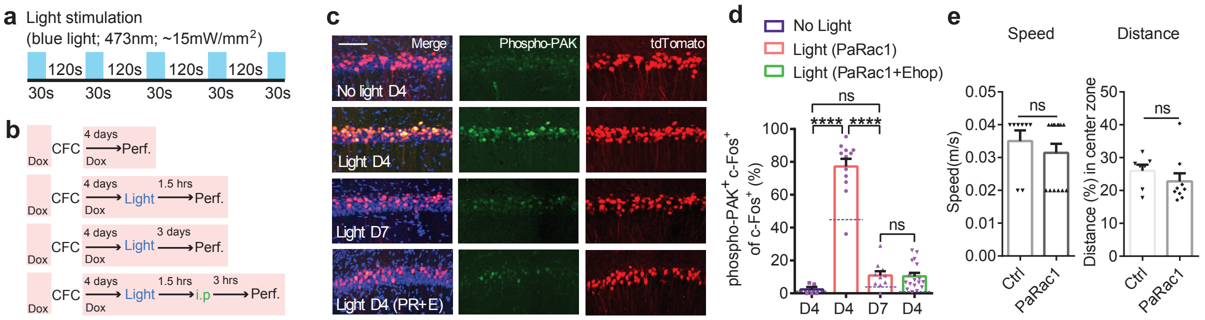

Supplementary Figure 6. The effects of optogenetic manipulation on the levels of phospho-PAK and the basic parameters of mice behavior. **a** A blue light from a laser was ON for 30 s to stimulate the photoactivatable Rac1, with trial intervals of 120 s (same light stimulating protocol used in immunohistochemistry). **b, c** Experimental schedule (b) and representative immunohistochemistry of phospho-PAK (green) in coronal section of CA1 engram cells with PaRac1-tdTomato expression (c). When off Dox for 2 days, mice were subjected to CFC and then mice were put back on Dox. These trained mice were killed after the following treatments: without light stimulation (No light D4); 1.5 hr followed by light stimulation (Light D4); 3 days followed by light stimulation (Light D7); 3 hr followed by Ehop016 injection (Light D4 PaRac1+Ehop016 (PR+E)). Phospho-PAK (green) is detected in the CA1 engram cells in light stimulated or no light stimulated animals. Arrows point at examples of phospho-PAK in PaRac1 expressing engram cells. Scale bar, 100 µm. Perfusion represent as Perf.. **d** Percentages of CA1 engram cells labeling with anti-phospho-PAK. Chance levels, indicated by grape dashed lines, were estimated at 1.53% (No light D4), 42.35% (Light D4 PaRac1), 2.56% (Light D7 PaRac1) and 0.6% (Light D4 PaRac1+Ehop016). ****$P < 0.0001$ (one-way ANOVA); n = 5-17 slices from 5 mice for each group. **e** Optogenetic manipulation had no effects on the basic parameters of mice behavior, including locomotor activity and anxiety, as indicated by both the speed and distance in the arena. n = 8, 14 mice for speed and n = 7, 9 mice for distance (Unpaired t-test). Data are presented as means ± SEM. Also see Supplementary Table 1.

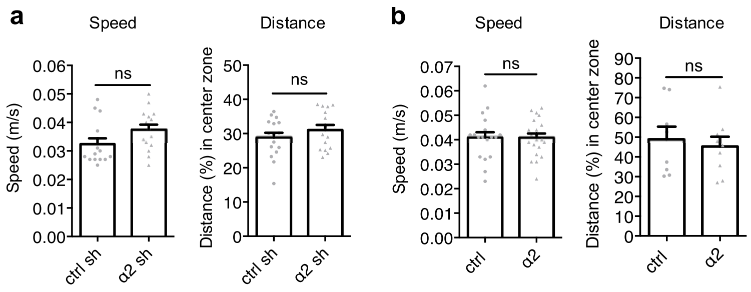

Supplementary Figure 7. α2-chimaerin knockdown and overexpression had no effects on the basic parameters of mice behaviors one-shock CFC. **a** and **b** Viral injections had no effects on the basic parameters of mice behaviors, including locomotor activity and anxiety, as indicated by both the speed and distance in the arena. *n* = 14, 15 mice (a) (Unpaired t-test), *n* = 18, 20 mice (b) for speed and *n* = 8, 9 mice for distance (Unpaired t-test). Data are presented as means ± SEM. Also see Supplementary Table 1.

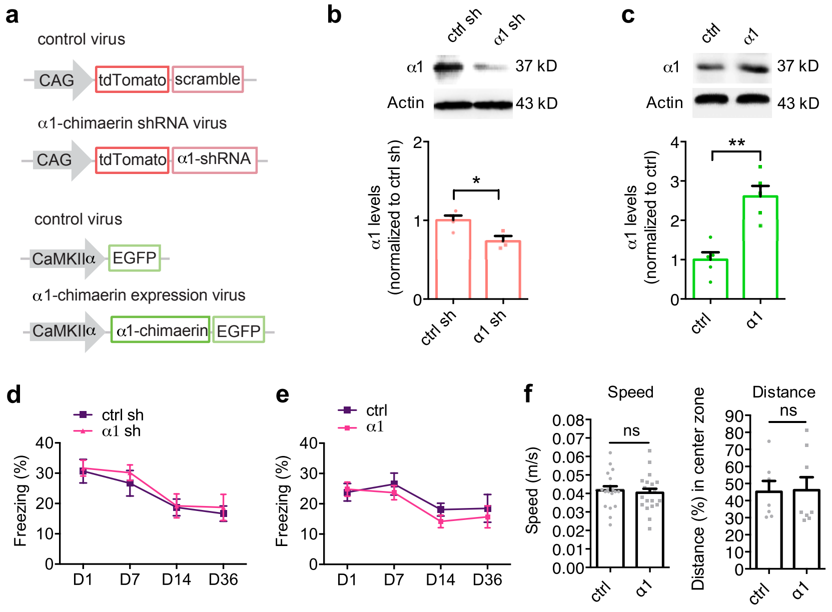

Supplementary Figure 8. α1-chimaerin knockdown and overexpression of had no effects on either the basic parameters of mice behavior or the memory retention curve. **a** Diagram of the AAV expressing either a short hairpin RNA (shRNA) sequence (Buttery et al., 2006) targeting α1-chimaerin (AAV-α1- shRNA) fused to Tandem dimer Tomato (tdTomato) under the control of the CMV early enhancer/chicken β actin (CAG) promoter or the α1-chimaerin (AAV-α1-chimaerin) fused to EGFP under the control of the calcium/calmodulin-dependent protein kinase IIα (CaMKIIα) promoter. **b** and **c** Representative western blots (top) and data (bottom) illustrating the levels of α1-chimaerin in mice injected with either AAV-α1 shRNA (α1 sh) or AAV-α1-chimaerin (α1) in the hippocampus. *$P < 0.05$, and **$P < 0.01$ (from Unpaired t-test); $n = 4$, 3 mice (b), $n = 3$, 3 mice mice (c). **d** and **e** Time courses of mice freezing behavior. n = 8, 6, 8, 8, 13, 10, 14, 15 mice (d) and n = 8, 8, 6, 6, 17, 7, 11, 11 mice (e) (two-way ANOVA). **f** Effects of α1-chimaerin manipulation on the basic parameters of mice behavior. n = 19, 20 mice for speed and n = 7, 8 mice for distance (Unpaired t-test). Data are presented as means ± SEM. Also see Supplementary Table 1.

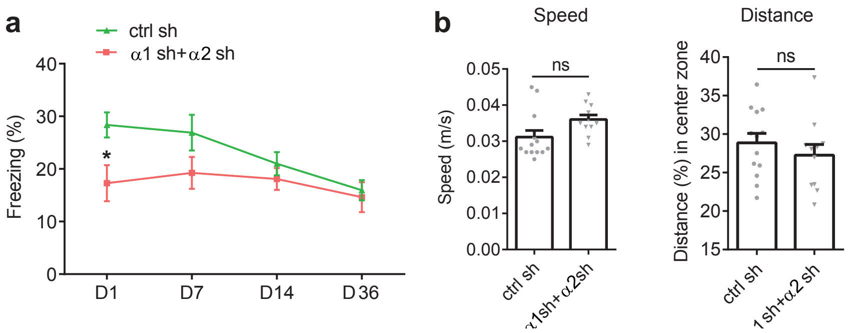

Supplementary Figure 9. The effects of double knockdown of α1-chimaerin and α2-chimaerin on the fear memory and the basic parameters of mice behavior. **a** Time courses of the freezing behavior. $*P < 0.05$ (from two-way ANOVA); $n = 15, 11, 13, 16, 11, 9, 15, 11$ mice. **b** AAV injection had no effects on the basic parameters of mice behaviors, including locomotor activity and anxiety, as indicated by the speed and distance in the arena. $n = 13, 11$ mice for speed and distance (Unpaired t-test). Data are presented as means ± SEM. Also see Supplementary Table 1.

Supplementary Table 1

Figure 1		Sample size (figure order)	Statistical Analysis	Treatment effect		Significance
a	time courses of freezing after one-shock CFC	*n* = 41, 28,17, 22, 21 mice	one-way ANOVA	F (4, 124) = 2.403	*P* = 0.0014	*P* < 0.01
	D1 vs 1 h		Bonferroni's multiple comparisons test		*P* > 0.9999	n.s.
	D1 vs D7		Bonferroni's multiple comparisons test		*P* > 0.9999	n.s.
	D1 vs D14		Bonferroni's multiple comparisons test		*P* = 0.0374	*P* < 0.05
	D1 vs D36		Bonferroni's multiple comparisons test		*P* = 0.0098	*P* < 0.01
b	protein levels of Rac1 activity after one-shock CFC	*n* = 12, 7, 7, 3, 4, 4, 4 mice	one-way ANOVA	F (7, 38) = 4.448	*P* = 0.0011	*P* < 0.01
	Na vs 1 h		Dunnett's multiple comparisons test		*P* = 0.955	n.s.
	Na vs D1		Dunnett's multiple comparisons test		*P* = 0.0138	*P* < 0.05
	Na vs D2		Dunnett's multiple comparisons test		*P* = 0.0126	*P* < 0.05
	Na vs D7		Dunnett's multiple comparisons test		*P* = 0.0488	*P* < 0.05

	Na vs D11		Dunnett's multiple comparisons test		*P* = 0.007	*P* < 0.01
	Na vs D14		Dunnett's multiple comparisons test		*P* > 0.9999	n.s.
c	Rac1 activity in CA1 after one-shock CFC	*n* = 15-17 slices from 7 mice for each group	one-way ANOVA	F (5, 114) = 3.956	*P* = 3.956	n.s.
	Na vs D1		Dunnett's multiple comparisons test		*P* < 0.0001	
	Na vs D2		Dunnett's multiple comparisons test		*P* < 0.0001	
	Na vs D7		Dunnett's multiple comparisons test		*P* < 0.0001	
	Na vs D11		Dunnett's multiple comparisons test		*P* < 0.0001	
	Na vs D14		Dunnett's multiple comparisons test		*P* = 0.6843	n.s.
d	1 h and 24 h memory	*n* = 8, 10, 6, 7, 4, 10 mice	two-way ANOVA	F (2, 39) = 7.309	*P* = 0.002	*P* < 0.01
	Ctrl vs Rac1-CA at 1 h		Sidak's multiple comparisons test		*P* = 0.8974	n.s.
	Ctrl vs Rac1-DN at 1 h		Sidak's multiple comparisons test		*P* = 0.9157	n.s.
	Ctrl vs Rac1-CA at 24 h		Sidak's multiple comparisons test		*P* = 0.0151	*P* < 0.05
	Ctrl vs Rac1-DN at 24 h		Sidak's multiple comparisons test		*P* = 0.0011	*P* < 0.01
e	memory retention curve	*n* = 10, 14 mice	two-way ANOVA	F (3, 76) = 2.857	*P* = 0.0425	*P* < 0.05

	Saline vs Ehop016 D1		Sidak's multiple comparisons test		*P* = 0.9483	n.s.
	Saline vs Ehop016 D4		Sidak's multiple comparisons test		*P* = 0.0392	*P* < 0.05
	Saline vs Ehop016 D7		Sidak's multiple comparisons test		*P* = 0.0195	*P* < 0.05
	Saline vs Ehop016 D14		Sidak's multiple comparisons test		*P* < 0.0001	
**Figure 2**						
d	c-fos$^+$ of DAPI (%)	*n* = 10, 9, 14 mice	one-way ANOVA	F (2, 30) = 5.728	*P* = 0.0078	n.s.
	CFC 24h vs Context		Dunnett's multiple comparisons test		*P* = 0.0015	*P* < 0.01
	CFC 24h vs Shock		Dunnett's multiple comparisons test		*P* < 0.0001	
e	Rac1-GTP$^+$ c-fos$^+$ of c-fos$^+$ (%)	*n* = 10, 9, 14 mice	one-way ANOVA	F (2, 30) = 2.984	*P* < 0.0001	
	CFC 24h vs Context		Dunnett's multiple comparisons test		*P* < 0.0001	
	CFC 24h vs Shock		Dunnett's multiple comparisons test		*P* < 0.0001	
g	memory retention curve	*n* = 11, 17, 15, 11, 6, 12, 13, 13 mice	two-way ANOVA	F (3, 90) = 2.549	*P* = 0.0607	n.s.
	Ctrl vs PaRac1 D1		Sidak's multiple comparisons test		*P* = 0.9816	n.s.
	Ctrl vs PaRac1 D4		Sidak's multiple comparisons test		*P* = 0.0009	*P* < 0.001
	Ctrl vs PaRac1 D7		Sidak's multiple comparisons test		*P* = 0.8135	n.s.

	Ctrl vs PaRac1 D14		Sidak's multiple comparisons test		*P* = 0.9997	n.s.
h	memory retention curve	*n* = 9, 5, 10, 7, 12, 6 mice	two-way ANOVA	F (2, 43) = 9.312	*P* = 0.0004	*P < 0.001*
	Ctrl+Saline vs PaRac1+Ehop016 Test 1		Tukey's multiple comparisons test		*P* = 0.0276	*P < 0.05*
	Ctrl+Saline vs PaRac1+Saline Test 1		Tukey's multiple comparisons test		*P* = 0.0131	*P < 0.05*
	PaRac1+Ehop016 vs PaRac1+Saline Test 1		Tukey's multiple comparisons test		P = 0.9811	n.s.
	Ctrl+Saline vs PaRac1+Ehop016 Test 2		Tukey's multiple comparisons test		*P* = 0.0658	n.s.
	Ctrl+Saline vs PaRac1+Saline Test 2		Tukey's multiple comparisons test		*P* = 0.0497	*P < 0.05*
	PaRac1+Ehop016 vs PaRac1+Saline Test 2		Tukey's multiple comparisons test		*P < 0.0001*	
i	memory retention	*n* = 9, 5, 8, 8 mice	two-way ANOVA	F (1, 26) = 0.04867	*P* = 0.8271	n.s.
	Ctrl+Saline vs PaRac1+Ehop016 Test 1		Bonferroni's multiple comparisons test		*P* = 0.7925	n.s.
	Ctrl+Saline vs PaRac1+Ehop016 Test 2		Bonferroni's multiple comparisons test		*P* > 0.9999	n.s.
j	memory retention curve	*n* = 4, 4, 4, 15, 11, 7 mice	two-way ANOVA	F (2, 42) = 4.756	*P* = 0.0138	n.s.
	Saline vs Ehop016 D14		Bonferroni's multiple comparisons test		*P* > 0.9999	n.s.
	Saline vs Ehop016 D15		Bonferroni's multiple comparisons test		*P* > 0.9999	n.s.

	Saline vs Ehop016 D22		Bonferroni's multiple comparisons test		*P* = 0.0009	*P* < 0.001
**Figure 3**						
a	protein level of α2-chimaerin after one-shock CFC	*n* = 12, 4, 6, 5, 6, 5 mice	one-way ANOVA	F (6, 30) = 3.131	*P* = 0.0167	*P* < 0.05
	Na vs 1 h		Dunnett's multiple comparisons test		*P* = 0.9946	n.s.
	Na vs D1		Dunnett's multiple comparisons test		*P* = 0.016	*P* < 0.05
	Na vs D7		Dunnett's multiple comparisons test		*P* = 0.0326	*P* < 0.05
	Na vs D11		Dunnett's multiple comparisons test		*P* = 0.9439	n.s.
	Na vs D14		Dunnett's multiple comparisons test		*P* = 0.9386	n.s.
b	protein level of α2-chimaerin	*n* = 4, 4, 4 mice	one-way ANOVA			
	Na vs CFC		Tukeyt's multiple comparisons test		*P* = 0.0294	*P* < 0.05
	Na vs CFC+ANI		Tukeyt's multiple comparisons test		*P* = 0.9745	n.s.
	CFC vs CFC+ANI		Tukeyt's multiple comparisons test		*P* = 0.0413	*P < 0.05*
**Figure 4**						
e and f	protein levels of α2-chimaerin					
	ctrl sh vs α2 sh	*n* = 3, 3 mice	Unpaired t-test (two-tailed)	t = 4.687 df = 4	*P* = 0.0094	*P* < 0.01
	ctrl vs α2	*n* = 7, 7 mice	Unpaired t-test (two-tailed)	t = 4.563 df = 12	*P* = 0.0007	*P* < 0.001

g and h	protein level of Rac1 activity					
	ctrl sh vs α2 sh	*n* = 3, 3 mice	Unpaired t-test (two-tailed)	t = 4.134 df = 4	*P* = 0.0144	*P* < 0.05
	ctrl vs α2	*n* = 7, 7 mice	Unpaired t-test (two-tailed)	t = 5.24 df = 12	*P* = 0.0002	*P* < 0.001
i	protein levels of Rac1 activity					
	CFC vs α2 sh+CFC	*n* = 3, 4 mice	Unpaired t-test (two-tailed)	t = 3.158 df = 5	*P* = 0.0251	*P* < 0.05
j	memory retention curve	*n* = 7, 8, 15, 13, 11, 11, 16, 10, 9, 11 mice	two-way ANOVA	F (4, 101) = 2.690	*P* = 0.0353	*P* < 0.05
	ctrl sh vs α2 sh 1h		Dunnett's multiple comparisons test		*P* = 0.9989	n.s.
	ctrl sh vs α2 sh D1		Dunnett's multiple comparisons test		*P* = 0.0030	*P* < 0.01
	ctrl sh vs α2 sh D7		Dunnett's multiple comparisons test		*P* = 0.0211	*P* < 0.05
	ctrl sh vs α2 sh D14		Dunnett's multiple comparisons test		*P* > 0.9999	n.s.
	ctrl sh vs α2 sh D36		Dunnett's multiple comparisons test		*P* = 0.9993	n.s.
k	memory retention curve	*n* = 11, 13, 11, 13, 12, 7 mice	two-way ANOVA	F (2, 61) = 2.139	*P* = 0.1265	n.s.
	ctrl vs α2 1h		Dunnett's multiple comparisons test		*P* = 0.9753	n.s.
	ctrl vs α2 D1		Dunnett's multiple comparisons test		*P* = 0.0301	*P* < 0.05
	ctrl vs α2 D14		Dunnett's multiple comparisons test		*P* = 0.0192	*P* < 0.05

l	memory retention curve	*n* =3, 10, 10, 8, 12, 12, 5, 7, 9 mice	two-way ANOVA	F (4, 67) = 5.095	*P* = 0.0012	*P* < 0.05
	ctrl sh+Saline vs α2 sh+Ehop016 1 h		Tukeyt's multiple comparisons test		*P* = 0.9681	n.s.
	ctrl sh+Saline vs α2 sh 1 h		Tukeyt's multiple comparisons test		*P* = 0.9982	n.s.
	α2 sh vs ctrl sh+Saline D1		Tukeyt's multiple comparisons test		*P* = 0.0038	*P* < 0.01
	ctrl sh+Saline vs α2 sh+Ehop016 D1		Tukeyt's multiple comparisons test		*P* = 0.0030	*P* < 0.01
	α2 sh vs ctrl sh+Saline D14		Tukeyt's multiple comparisons test		*P* = 0.9828	n.s.
	ctrl sh+Saline vs α2 sh+Ehop016 D14		Tukeyt's multiple comparisons test		*P* < 0.0001	
**Figure 5**						
a	social discrimination index					
	ctrl sh vs α2 sh	*n* = 13, 13 mice	Unpaired t-test (two-tailed)	t = 2.771 df = 24	*P* = 0.0106	*P* < 0.05
b	novel object recognition	*n* = 12, 12 mice	two-way ANOVA	F (1 , 44) = 1.921	*P* = 0.1727	n.s.
	ctrl sh vs α2 sh 1 h		Sidak's multiple comparisons test		*P* = 0.9157	n.s.
	ctrl sh vs α2 sh 24 h		Sidak's multiple comparisons test		*P* = 0.0477	*P* < 0.05
**Figure 6**	LTP					
a	slice injected with ctrl shNA vs slice injected with AAV-α2 shNA	*n* = 8, 9 mice	Unpaired t-test (two-tailed)	t = 36.39 df = 40	*P* < 0.0001	
b	slice injected with ctrl vs slice injected with AAV-α2-chimaerin	*n* = 11, 8 mice	Unpaired t-test (two-tailed)	t = 152.5 df = 42	*P* < 0.0001	
**Figure s1**						

a	time courses of freezing after thee-shock CFC	*n* = 8, 48, 35, 45, 29 mice	one-way ANOVA	$F (4, 160) = 0.7659$	$P = 0.5489$	n.s.
	D1 vs 1 h		Dunnett's multiple comparisons test		$P = 0.7259$	n.s.
	D1 vs D7		Dunnett's multiple comparisons test		$P = 0.5762$	n.s.
	D1 vs D14		Dunnett's multiple comparisons test		$P = 0.7757$	n.s.
	D1 vs D36		Dunnett's multiple comparisons test		$P = 0.3465$	n.s.
**Figure s2**						
a	protein levels of Rac1 activity after context-only or shock-only	*n* = 3, 3, 4 mice	one-way ANOVA	$F (2, 7) = 0.1845$	$P = 0.8355$	n.s.
	Naive vs Context		Dunnett's multiple comparisons test		$P = 0.8393$	n.s.
	Naive vs Shock		Dunnett's multiple comparisons test		$P = 0.9996$	n.s.
b	protein levels of α2-chimaerin after context or shock	*n* = 3, 3, 3 mice	one-way ANOVA	$F (2, 6) = 0.4154$	$P = 0.6777$	n.s.
	Naive vs Context		Dunnett's multiple comparisons test		$P = 0.5935$	n.s.
	Naive vs Shock		Dunnett's multiple comparisons test		$P = 0.9064$	n.s.

c	protein levels of α1-chimaerin after one-shock CFC	*n* = 9, 8, 9 mice	one-way ANOVA	F (2, 23) = 0.1169	*P* = 0.8902	n.s.
	Na vs 1 h		Dunnett's multiple comparisons test		*P* = 0.8872	n.s.
	Na vs D1		Dunnett's multiple comparisons test		*P* > 0.9999	n.s.
d	protein levels of Rac1 activity after thee-shock CFC	*n* = 3, 3, 3, 3 mice				
	Na vs 1 h		Unpaired t-test (two-tailed)	t = 2.894 df = 4	*P* = 0.0444	*P* < 0.05
	Na vs 4 h		Unpaired t-test (two-tailed)	t = 7.173 df = 4	*P* = 0.002	*P* < 0.01
	Na vs D1		Unpaired t-test (two-tailed)	t = 14.83 df = 4	*P* = 0.0001	
e	protein levels of α2-chimaerin after thee-shock CFC	*n* = 3, 3, 3, 3 mice	one-way ANOVA	F (3, 12) = 0.4034	*P* = 0.9896	n.s.
	Na vs 1 h		Dunnett's multiple comparisons test		*P* = 0.4363	n.s.
	Na vs 4 h		Dunnett's multiple comparisons test		*P* = 0.3039	n.s.
	Na vs D1		Dunnett's multiple comparisons test		*P* = 0.7878	n.s.

f	fear memory	*n* = 15, 15 mice	Unpaired t-test (two-tailed)	t = 0.08428 df = 28	*P* = 0.5095	n.s.
	ctrl sh vs α2 sh 24 h				*P* = 0.9334	n.s.
**Figure s3**						
c	protein level of Rac1 activity	*n* = 5, 5, 5 mice	one-way ANOVA	F (2, 12) = 1.219	*P* < 0.0001	
	Ctrl vs Rac1-DN		Dunnett's multiple comparisons test		*P* = 0.0024	*P* < 0.01
	Ctrl vs Rac1-CA		Dunnett's multiple comparisons test		*P* = 0.007	*P* < 0.01
e	protein level of Rac1 activity	*n* = 5, 5, 5 mice	one-way ANOVA	F (2, 12) = 0.1865	*P* = 0.5416	n.s.
	Ctrl vs Rac1-DN		Dunnett's multiple comparisons test		*P* = 0.6620	n.s.
	Ctrl vs Rac1-CA		Dunnett's multiple comparisons test		*P* = 0.9951	n.s.
**Figure s4**						
a	Saline vs Ehop016	*n* = 4, 4 mice	Unpaired t-test (two-tailed)	t = 6.770 df = 6	*P* = 0.0005	*P* < 0.001
b	Saline vs Ehop016 at 1 h	*n* = 4, 5, 5, 9 mice	Unpaired t-test (two-tailed)		*P* = 0.4119	n.s.
	Saline vs Ehop016 at 24 h		Unpaired t-test (two-tailed)		*P* = 0.0316	*P* < 0.05
e	basic parameters					
	Saline vs α2 sh+Ehop016 speed	*n* = 5, 5 mice	Unpaired t-test (two-tailed)	t = 0.5704 df = 8	*P* = 0.5841	n.s.

	Saline vs α2 sh+Ehop016 distance	*n* = 5, 5 mice	Unpaired t-test (two-tailed)	t = 0.2100 df = 8	*P* = 0.8389	n.s.
**Figure s5**						
b	c-fos$^-$ vs c-fos$^+$	*n* = 14, 14 mice	Unpaired t-test (two-tailed)	t = 15.45 df = 26	*P* < 0.0001	
**Figure s6**						
d	protein level of phospho-PAK	*n* = 5-17 slices from 5 mice for each group	one-way ANOVA	F (3, 40) = 1.539	*P* = 0.2193	n.s.
	D4 no light vs D4 light		Tukeyt's multiple comparisons test		*P* < 0.0001	
	D4 light vs D7 light		Tukeyt's multiple comparisons test		*P* < 0.0001	
	D4 no light vs D7 light		Tukeyt's multiple comparisons test		*P* = 0.4775	n.s.
	D7 light vs D4 light (PaRac1+Ehop)		Tukeyt's multiple comparisons test		*P* = 0.9994	n.s.
e	basic parameters					
	Ctrl vs PaRac1 speed	*n* = 8, 14 mice	Unpaired t-test (two-tailed)	t = 0.8116 df = 20	*P* = 0.4266	n.s.
	Ctrl vs PaRac1 distance	*n* = 7, 9 mice	Unpaired t-test (two-tailed)	t = 0.9862 df = 14	*P* = 0.3408	n.s.
**Figure s7**	basic parameters					
a	ctrl sh vs α2 sh speed	*n* = 14, 15 mice	Unpaired t-test (two-tailed)	t = 1.818 df = 27	*P* = 0.0802	n.s.
	ctrl sh vs α2 sh distance	*n* = 14, 15 mice	Unpaired t-test (two-tailed)	t = 0.5471 df = 27	*P* = 0.5888	n.s.

b	ctrl vs α2 speed	*n* = 18, 20 mice	Unpaired t-test (two-tailed)	t = 0.03188 df = 36	*P* = 0.9747	n.s.
	ctrl vs α2 distance	*n* = 8, 9 mice	Unpaired t-test (two-tailed)	t = 0.4337 df = 15	*P* = 0.6707	n.s.
**Figure s8**						
	protein levels of α1-chimaerin					
b	ctrl sh vs α1 sh	*n* = 4, 3 mice	Unpaired t-test (two-tailed)		*P* = 0.0324	*P* < 0.05
c	ctrl vs α1	*n* = 3, 3 mice	Unpaired t-test (two-tailed)		*P* = 0.0094	*P* < 0.01
d	memory retention curve after CFC in α1 sh mice	*n* = 8, 6, 8, 8, 13, 10, 14, 15 mice	two-way ANOVA	F (3, 74) = 0.05223	*P* = 0.9841	n.s.
	ctrl sh vs α1 sh D1		Bonferroni's multiple comparisons test		*P* > 0.9999	n.s.
	ctrl sh vs α1 sh D7		Bonferroni's multiple comparisons test		*P* > 0.9999	n.s.
	ctrl sh vs α1 sh D14		Bonferroni's multiple comparisons test		*P* > 0.9999	n.s.
	ctrl sh vs α1 sh D36		Bonferroni's multiple comparisons test		*P* > 0.9999	n.s.
e	memory retention curve after CFC in α1 mice	*n* = 8, 8, 6, 6, 17, 7, 11, 11 mice	two-way ANOVA	F (3, 66) = 0.2604	*P* = 0.8536	n.s.
	ctrl vs α1 D1		Bonferroni's multiple comparisons test		*P* > 0.9999	n.s.

	ctrl vs α1 D7		Bonferroni's multiple comparisons test		*P* > 0.9999	n.s.
	ctrl vs α1 D14		Bonferroni's multiple comparisons test		*P* > 0.9999	n.s.
	ctrl vs α1 D36		Bonferroni's multiple comparisons test		*P* > 0.9999	n.s.
f	ctrl vs α1 speed	*n* = 19, 20 mice	Unpaired t-test (two-tailed)	t = 0.4243 df = 37	*P* = 0.6738	n.s.
	ctrl vs α1 distance	*n* = 7, 8 mice	Unpaired t-test (two-tailed)	t = 0.09863 df = 13	*P* = 0.9229	n.s.
**Figure s9**						
a	memory retention curve after one-shock CFC	*n* = 15, 11, 13, 16, 11, 9, 15, 11 mice	two-way ANOVA	F (3, 93) = 1.494	*P* = 0.2213	n.s.
	ctrl sh vs α1 sh+α2 sh D1		Bonferroni's multiple comparisons test		*P* = 0.013	*P* < 0.05
	ctrl sh vs α1 sh+α2 sh D7		Bonferroni's multiple comparisons test		*P* = 0.2726	n.s.
	ctrl sh vs α1 sh+α2 sh D14		Bonferroni's multiple comparisons test		*P* > 0.9999	n.s.
	ctrl sh vs α1 sh+α2 sh D36		Bonferroni's multiple comparisons test		*P* > 0.9999	n.s.
b	speed of basic parameters		one-way ANOVA	F (2, 36) =2.220	*P* = 0.1233	n.s.
	ctrl sh vs α1 sh+α2 sh	*n* = 13, 11 mice	Dunnett's multiple comparisons test		*P* = 0.1915	n.s.
	distance travelled		one-way ANOVA	F (2, 36) =0.4895	*P* = 0.6169	n.s.

	ctrl sh vs α1 sh+α2 sh	*n* = 13, 11 mice	Dunnett's multiple comparisons test		*P* = 0.5992	n.s.
